# Pediatric Post‐Vaccine Aluminum Granuloma: Morin Stain as a Diagnostic Aid

**DOI:** 10.1111/cup.14797

**Published:** 2025-02-24

**Authors:** Justin R. Chang, Alvin Wong, Julio A. Diaz‐Perez, Chunyu Cai, Mark C. Mochel

**Affiliations:** ^1^ Department of Pathology Virginia Commonwealth University Health System Richmond Virginia USA; ^2^ Department of Surgery Virginia Commonwealth University Health System Richmond Virginia USA; ^3^ Department of Dermatology Virginia Commonwealth University Health System Richmond Virginia USA; ^4^ Department of Pathology University of Texas Southwestern Dallas Texas USA

**Keywords:** aluminum, granuloma, vaccine

## 1 Introduction

Infrequently, vaccine injection sites become chronically indurated, a phenomenon spanning a wide age range, occurring 7 months to 8 years post‐vaccination, and occasionally persisting for years [1, 2]. Excision is curative. Histopathologic examination typically reveals subcutaneous granulomatous inflammation with histiocytes containing granular amphophilic cytoplasm, a finding correlated with the presence of aluminum salts, an adjuvant component of many vaccines. Ancillary tests to confirm the presence of aluminum include energy‐dispersive x‐ray microanalysis and, more practical for histology laboratories, histochemical studies such as the morin stain, which forms a green fluorescent complex with aluminum [3]. Here, we present a case of a persistent nodular vaccine‐site reaction in a child with characteristic histopathologic findings and the use of a morin stain to detect the presence and distribution of aluminum.

## 2 Case

**FIGURE 1:**
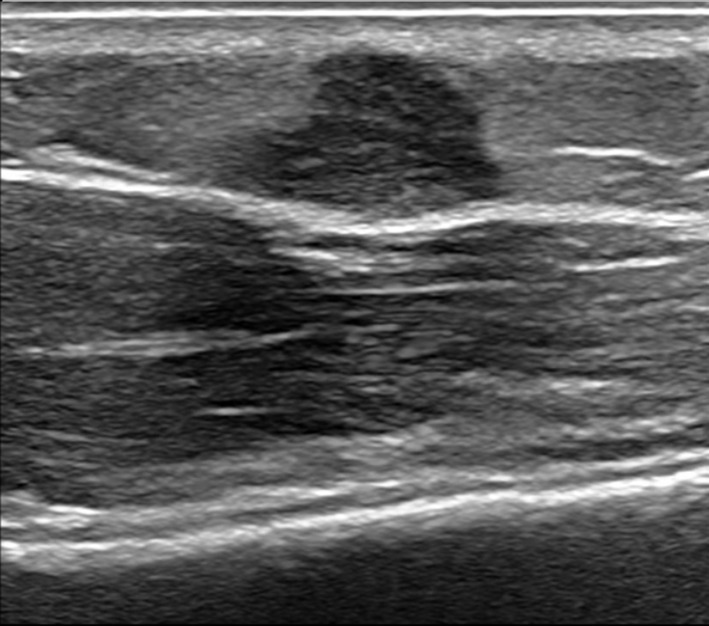
An ultrasound examination of the left deltoid nodule at the vaccination site revealed a lobulated, hypoechoic lesion measuring 0.8 cm.

A 22‐month‐old male with a history of mild atopic dermatitis and pityriasis rosea presented with an enlarging nodule in the left shoulder following vaccinations at 8 months of age. DTaP‐IPV‐HepB (Diphtheria, Tetanus, Pertussis, Poliovirus, hepatitis B), Pneumococcal conjugate, and *Haemophilus influenzae* type B vaccines had been administered at the site of the lesion. Physical examination revealed a firm subcutaneous nodule, approximately 1 cm in greatest dimension, overlying the left deltoid muscle with an overlying mildly hypertrophic scar. An ultrasound study revealed a 0.8 cm lobulated hypoechoic lesion (Figure 1). While the lesion was suspected to be a vaccine reaction, the patient's family opted for removal of the lesion for definitive diagnosis.

**FIGURE 2:**
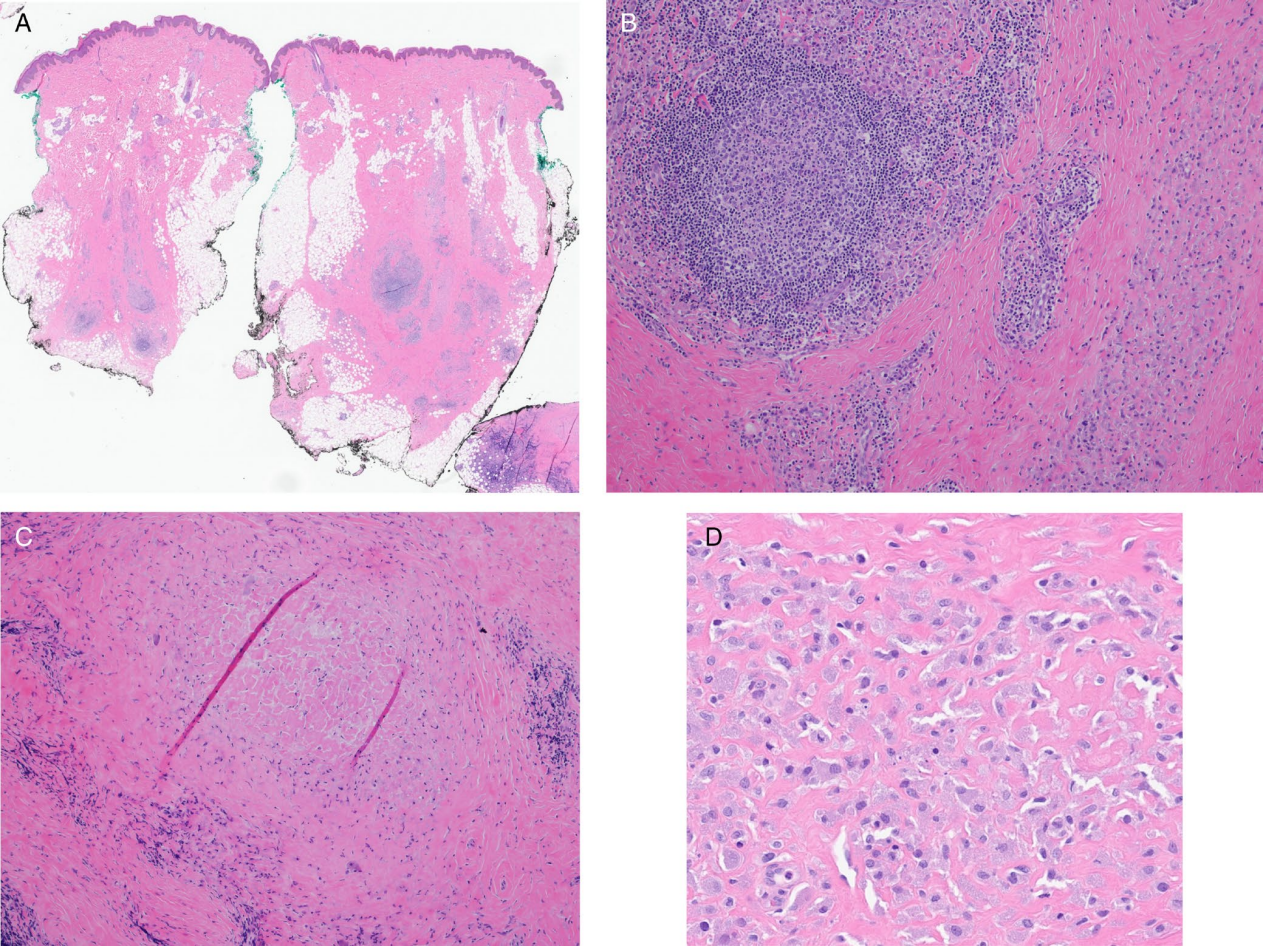
Biopsy of skin and subcutis (A, 10×) with subcutaneous fibrosis containing patchy lymphohistiocytic inflammation with a few lymphoid follicles (B, 100×), palisading granulomatous inflammation about altered collagen (C, 100×) and loosely aggregated histiocytes with granular amphophilic cytoplasm (D, 400×).

**FIGURE 3:**
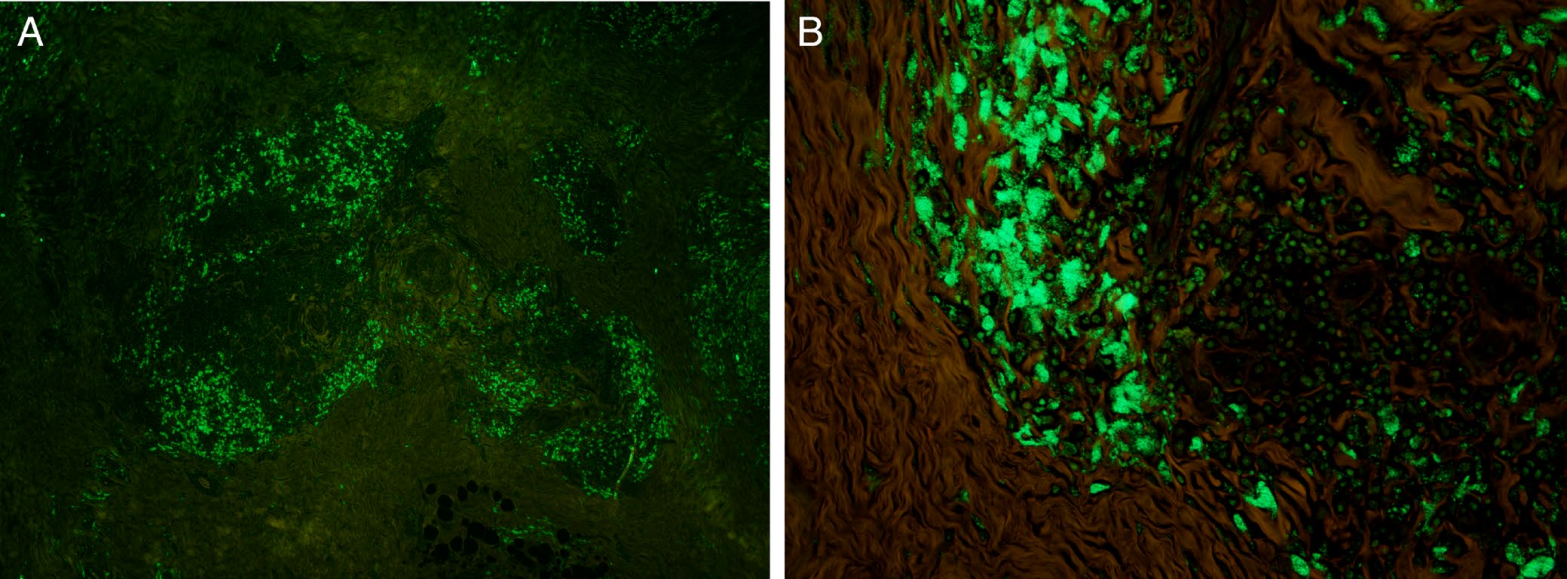
Morin stain displays green fluorescent positivity (A, 10×), indicating the presence of aluminum and corresponding to the granular cytoplasm of the granular histiocytes (B, 400×).

Histopathological examination of the excision specimen revealed subcutaneous fibrosis with patchy lymphohistiocytic inflammation, scattered lymphoid follicles, and focal palisaded granuloma surrounding altered collagen, which contained rare purplish non‐polarizable material (Figure 2A–C). Histiocytes had granular amphophilic cytoplasm (Figure 2D). A Grocott methenamine silver stain was negative for fungi. Ziehl‐Neelsen and Fite stains were negative for mycobacteria. The histopathologic features were consistent with a persistent vaccine reaction, possibly related to aluminum adjuvants. Subsequently, a morin stain was prepared, as described previously [3], for the specific identification of aluminum. Through the green channel in fluorescence microscopy, aluminum granules were seen (Figure 3A), mostly within the cytoplasm of the granular macrophages (Figure 3B). Taken together, the histopathologic and histochemical findings were consistent with a vaccine‐site reaction secondary to aluminum salt.

## 3 Discussion

Given that vaccine‐site reactions typically resolve without intervention, biopsy is rarely performed. Consequently, few studies have detailed the histopathology of reactions to aluminum‐adjuvant‐containing vaccines. Affected subcutaneous tissue typically exhibits lymphohistiocytic inflammation, accompanied by varying degrees of fat necrosis and fibrosis [1, 2, 3, 4]. Variable features include granulomatous inflammation and prominent lymphoid follicles, sometimes with germinal centers. Associated granulomas may be necrotizing or palisading with associated degenerative collagen. When dense, the lymphoid infiltrates may raise suspicion for follicular lymphoma or marginal zone lymphoma [1, 4]. The dermis may contain perivascular and periadnexal lymphocytes; these features, when paired with lobular panniculitis and lymphoid infiltrates, may mimic lupus panniculitis [1]. A characteristic feature is the presence of loosely aggregated histiocytes with purplish cytoplasmic granules (Figure 2D). This granular cytoplasm is reportedly positive on Giemsa and periodic acid‐Schiff (PAS) stains, while scanning electron microscopy of such histiocytes reveals crystalline material [1].

Energy‐dispersive x‐ray microanalysis, a specialized technique utilizing electron microscopy and x‐ray diffraction, has been used to confirm the presence of aluminum in vaccine site reactions [1, 3, 5]. However, this technique requires separate tissue preparations, specialized laboratory equipment, and rare expertise. Histochemical methods, such as the ammonium aurintricarboxylate stain [6] and the morin stain, are, in contrast, more accessible to anatomic pathology laboratories.

As previously described [3], morin, a flavonoid extracted from *Psidium guajava* (common guava), binds aluminum, thereby creating a fluorescent complex detectable by fluorescence microscopy (Figure 3A,B) [3]. The morin stain has demonstrated sensitivity and specificity for identifying aluminum deposits in formalin‐fixed, paraffin‐embedded sections [3], and may be valuable in cases where an adult patient's vaccination history is unclear or remote.

To the authors' knowledge, the dermatopathology literature contains only two prior studies utilizing the morin stain, both in the context of post‐vaccine cutaneous lymphoid hyperplasia. In 2004, a study reported 10 cases of cutaneous lymphocytic hyperplasia presenting as subcutaneous nodules following various vaccinations. Histopathology revealed lymphocytic inflammation, fibrosis, and granulomas, with the aluminum detection via morin stain in six cases [7]. In 2005, another study utilized morin stains to detect aluminum hydroxide in post‐vaccine nodules of nine patients who had received hepatitis A and B vaccines. Histopathology showed dense lymphoid infiltrates with admixed granular histiocytes, while morin stain revealed green‐fluorescent cytoplasmic granules, correlating with findings from electron probe microanalysis, which identified aluminum crystals [8]. More recently, morin stains have helped diagnose macrophagic myofasciitis, a post‐vaccine condition requiring the detection of aluminum within macrophages [3]. This series also included one subcutaneous post‐vaccine granuloma.

Although morin can also complex other metals and metalloids, including zinc, boron, and beryllium, vaccine adjuvants reportedly do not contain these elements [3]. In our experience, the stain is accessible, requiring a straightforward histochemical protocol, previously described in detail [3], which utilizes common laboratory reagents and the relatively inexpensive morin reagent [9], ultimately costing approximately the same as a PAS stain. However, interpretation requires an immunofluorescence microscope.

Potential alternative techniques for the detection of aluminum include the PAS stain and Epstein–Barr encoding region (EBER) in situ hybridization (ISH). One series on vaccine reactions [1] and rare case reports of post‐biopsy aluminum chloride [10, 11] have documented that PAS stains may highlight foci of aluminum salts, although negative controls were not reported. PAS stains highlight macrophages containing various polysaccharides, glycoproteins, and other substances, raising challenges with the specificity for PAS‐positive macrophages. EBER ISH has been noted to stain post‐vaccine aluminum deposits idiosyncratically [2], although one group found hemostatic aluminum deposits to be EBER‐negative [12]. However, EBER ISH stains genuine viral particles in affected nuclei, potentially raising diagnostic challenges between true and idiosyncratic EBER positivity. We note that the literature for PAS and EBER ISH for detection of aluminum is emerging and, to date, is limited to case reports and a small series without complete accounting of sensitivity and specificity [2, 10, 11]. In contrast, the morin stain has demonstrated high specificity for the detection of aluminum deposits across several studies, some with negative controls [3, 7, 8].

While careful histopathologic examination and clinical correlation usually permit the diagnosis of vaccination‐site reactions, the confirmation of aluminum deposits may assist in the diagnosis. We argue that the morin stain is an accessible and specific method of identifying and localizing aluminum salt deposits in tissue, thereby enabling a diagnosis of persistent vaccine reaction.

## Ethics Statement

The authors have nothing to report.

## Conflicts of Interest

The authors declare no conflicts of interest.

## Data Availability

Data sharing is not applicable to this article as no new data were created or analyzed in this study.
